# Mesenchymal Stem Cells Subpopulations: Application for Orthopedic Regenerative Medicine

**DOI:** 10.1155/2016/3187491

**Published:** 2016-09-20

**Authors:** Vanessa Pérez-Silos, Alberto Camacho-Morales, Lizeth Fuentes-Mera

**Affiliations:** ^1^Departamento de Bioquímica y Medicina Molecular, Universidad Autónoma de Nuevo León (UANL), Monterrey, NL, Mexico; ^2^Unidad de Terapias Experimentales, Centro de Investigación y Desarrollo en Ciencias de la Salud, Universidad Autónoma de Nuevo León (UANL), Monterrey, NL, Mexico; ^3^Unidad de Neurociencias, Centro de Investigación y Desarrollo en Ciencias de la Salud, Universidad Autónoma de Nuevo León (UANL), Monterrey, NL, Mexico

## Abstract

Research on mesenchymal stem cells (MSCs) continues to progress rapidly. Nevertheless, the field faces several challenges, such as inherent cell heterogeneity and the absence of unique MSCs markers. Due to MSCs' ability to differentiate into multiple tissues, these cells represent a promising tool for new cell-based therapies. However, for tissue engineering applications, it is critical to start with a well-defined cell population. Additionally, evidence that MSCs subpopulations may also feature distinct characteristics and regeneration potential has arisen. In this report, we present an overview of the identification of MSCs based on the expression of several surface markers and their current tissue sources. We review the use of MSCs subpopulations in recent years and the main methodologies that have addressed their isolation, and we emphasize the most-used surface markers for selection, isolation, and characterization. Next, we discuss the osteogenic and chondrogenic differentiation from MSCs subpopulations. We conclude that MSCs subpopulation selection is not a minor concern because each subpopulation has particular potential for promoting the differentiation into osteoblasts and chondrocytes. The accurate selection of the subpopulation advances possibilities suitable for preclinical and clinical studies and determines the safest and most efficacious regeneration process.

## 1. Introduction

Stem cells are well defined by their ability to self-renew and to differentiate into a range of cell types. In the adult organism, these cells are responsible for maintaining the homeostasis of their respective tissues. The maintenance of stemness and pluripotency of stem cells proceeds in the stem cell niche, where stem cells receive adequate signals from the stroma and other cell types either via receptors or by secreted soluble factors within this microenvironmental niche [1].

Mesenchymal stem cells (MSCs) were generally defined based on their capacity to self-renew and on their phenotype. The International Society for Cellular Therapy (ISCT) has proposed the following minimum criteria for the definition of the MSCs: (I) adherence to plastic surfaces under standard cell culture conditions; (II) the expression of cell surface markers, such as CD90, CD73, and CD105, and the lack of expression of CD14, CD34, CD45, CD79, or CD19 and HLA-DR, and (III) the capability to differentiate into chondrocytes, osteoblasts, and adipocytes [2].

Considerable effort has been expended to identify specific surface markers that characterize MSCs, yet disagreement in the literature has prevented the creation of definitive standards. In this regard, additional studies have also associated other markers with MSCs, such as CD271, Stro-1, vascular cell adhesion molecule-1 (VCAM-1), and CD146 [3–5].

The current review highlights recent findings in the identification and isolation of MSCs subpopulations, which could improve expansion strategies in the near future and the clinical use of MSCs differentiated into osteogenic and chondrogenic lineages.

MSCs subpopulations from several sources in conjunction with specific growth factors and/or scaffold are potentially useful for a variety of clinical orthopedic conditions involving bone and cartilage. There are several clinical trials using MSCs subpopulations to repair critical-sized injuries caused by trauma or infection, aside from replacing chronically degenerated tissue, such as articular cartilage. We recognize that variability in MSC-based clinical trial outcomes is likely due not only to differences amongst various MSCs sources but also to cell heterogeneity and inadequate selection of the subpopulation.

## 2. Sources of Mesenchymal Stem Cells

MSCs were first depicted by Friedenstein et al. in 1968 as adherent fibroblast-like cells with multipotent differentiation abilities. This study indicated that clonal populations belonging to the colony forming unit-fibroblastoids (CFU-Fs) result in osteoblasts, chondrocytes, and hematopoietic supportive cells* in vivo* [6]. MSCs were initially isolated from bone marrow (BM), and, in recent years, the isolation of adult mesenchymal stem cells from different sources has been reported. The comparative quality, character, and differentiation potential of MSCs from each of these sources differ and are still debated. MSCs have been isolated from multiple adult human tissues, such as adipose tissue [7, 8], articular cartilage [9], brain [10], endometrium [11], menstrual blood [12], peripheral blood [13], skin and foreskin [14, 15], and synovial fluid [16]. Additionally, perinatal organs and tissues that are normally discarded after delivery, namely, amniotic fluid [17], amniotic membrane [18, 19], full placenta and fetal membrane [20], subamniotic umbilical cord lining membrane [21, 22], and Wharton's jelly [23], have been shown to be rich sources of proliferative MSCs. Other sources include dental tissue, such as the pulp tissue of permanent human dental pulp stem cells (DPSCs) [24], stem cells from human exfoliated deciduous teeth (SHED) [25], periodontal ligament progenitor cells (PDLPs), and PDL stem cells (PDLSCs) [26]. Satellite cells in muscle and pericytes around blood vessels also share multipotent characteristics to differentiate into connective tissue phenotypes under specific conditions [27, 28].

Interestingly, in recent years, the use of bone marrow as a source of MSCs has decreased. A strong trend is observed in the use of various postfetal tissues besides adipose tissue as a major source for isolation.

## 3. Mesenchymal Stem Cell Subpopulations

MSCs were first identified in the bone marrow as an adherent population of nonhematopoietic stem cells with the capability of differentiating into different cell types of predominantly mesodermal origin. Cultures of MSCs show high heterogeneity, and the application of MSCs cultures in tissue regeneration depends mainly on their differentiation potential. Consequently, researchers are actively attempting to preselect cell subpopulations with higher osteochondrogenic potential in order to achieve a thorough translation of MSC-based therapies for orthopedic applications. Research over the last years has demonstrated that the use of a specific MSCs subpopulation ensures successful differentiation into a particular cell line.

MSCs are classically selected on the basis of their adherence to plastic, which however results in a heterogeneous population of cells. Prospective identification of the antigenic profile of the MSCs population (subpopulations) by FACS-based (fluorescence-activated cell sorting) approaches gives rise to cells with MSCs activity* in vitro* and would allow for the isolation of very pure populations of MSCs for research or clinical use [29, 30].

Several markers have been proposed to enrich these subpopulations, but the majority of these markers are defined for BM. In addition to a phenotypic variation depending on the MSCs source, the surface markers of freshly isolated MSCs may also differ from those of cultured MSCs. Although there have been attempts to increase MSCs purity by physical means, positive selection based on a specific MSCs marker offers a better alternative. Amongst a number of positive markers proposed in the past, CD271, CD105, CD44, CD90, and CD117 seem to offer adequate selectivity. Moreover, the isolation of homogeneous MSCs is best achieved by cell sorting with a combination of positive and negative markers.

**Figure 1:**
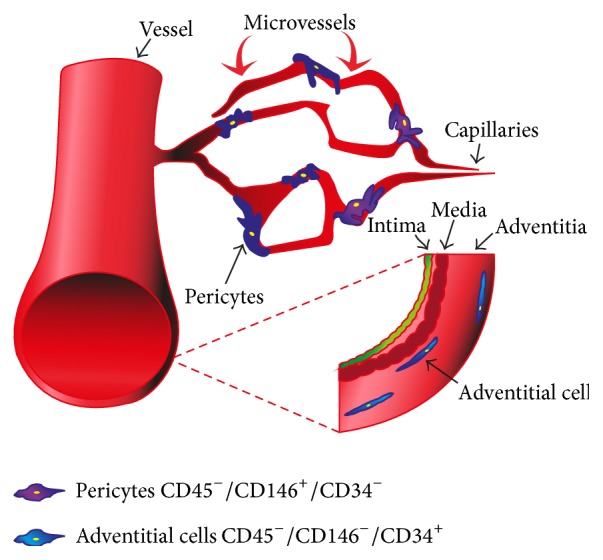
Pericytes and adventitial cells associated with skeletal muscle microvessels. A scheme showing the MSC subpopulations present in the three structural layers of blood vessels: pericytes (green) from media, adventitial cells (yellow) from adventitia, and myogenic endothelial cells from intima. Illustration of the phenotype of the corresponding cells: pericytes (CD45^−^/CD146^+^/CD34^−^) and adventitial cells (CD45^−^/CD146^−^/CD34^+^).

Blood vessels within skeletal muscle anchor several precursor populations. It is reported that pericytes, which surround endothelial cells of capillaries and venules, possess multipotent differentiation potential [28, 31]. In 2012, Corselli et al. reported that, in addition to MSCs being derived from pericytes, adventitial cells could also give rise to MSCs [32]. In a recent article by Zhao et al., it was demonstrated that, during incisor trauma, pericytes and adventitial cells (perivascular stem cells, PSC) are recruited to modulate hemostasis and repair. Further,* in vitro*, these PSC were shown to exhibit typical MSCs features. Sorting pericytes (CD45^−^/CD146^+^/CD34^−^) and adventitial cells (CD45^−^/CD146^−^/CD34^+^) by FACS is a process that requires a few hours [33–35] (Figure 1). This isolation method allows simultaneous purification of three multipotent cell populations, from three structural layers of blood vessels: pericytes from media, adventitial cells from adventitia, and myogenic endothelial cells from intima [36]. More recently, König et al. enriched a CD146^+^ subpopulation (CD146^+^/NG2^+^/CD45^−^) of pericytes from an isolated stromal vascular fraction of mouse fat tissue and demonstrated its efficient osteoblasts differentiation* in vitro* and ability to colonize cancellous bone scaffolds and regenerate large bone defects* in vivo* [37].

In a study conducted by Busser et al. (2015), immunomagnetic selections with 5 single surface markers were performed to isolate MSCs subpopulations from BM and adipose tissue (AT): CD271, SUSD2, MSCA-1, CD44, and CD34. Compared to the whole population of unselected ADSCs, the authors observed that CD271 selection can define AT cell population with higher multipotency and a higher proliferative capability [38].

Cuthbert et al. used FACS for the isolation of the subpopulation CD45^low^/CD73^+^/CD271^+^ from BM phenotype in order to enrich MSCs fractions. CD271^+^ immunomagnetic selection resulted in a substantial increase in MSCs purity and high expression of bone-related transcripts and vascularization, such as BMP-2, COL1A2, VEGFC, and SPARC transcripts [39].

Clearly, the use of strategies based on the coexpression of more than one surface marker improves the purity of the isolated MSCs population.

Mabuchi et al. (2013) reported in fresh human BM an improved clonal isolation technique and demonstrated that the combination of three cell surface markers (LNGFR, THY-1, and VCAM-1) allows the selection of highly enriched clonogenic cells. The marker combination LNGFR^+^/THY-1^+^/VCAM-1^+^ (LTV) represents a valuable strategy for the isolation of MSCs with broad potentiality features that are genetically more stable [40].

Likewise, based on the simultaneous use of three stem cell markers, Leyva-Leyva et al. (2013) selected and sorted by FACS two homogeneous subpopulations of hMSCs which coexpress the CD73^+^/CD44^+^/CD105^+^ (6%–12%) or CD73^+^/CD44^+^/CD105^−^ (80%–88%) antigens. This systematic method for the isolation of hMSCs generated homogeneous cultures for osteoblast differentiation with an enhanced ability to mineralize [18].

### 3.1. Osteogenic Differentiation from Mesenchymal Stem Cell Subpopulations

Unlike many other tissues, bone is an exceptional tissue that regenerates completely in the absence of scar tissue [41]. The bone healing process has three stages: inflammation, bone generation, and bone remodeling. When the bones fracture, bleeding occurs in the area resulting in inflammation and blood clotting at the fracture site. These events provide the primary structural stability and support for the production of new bone. Following an inflammatory stage, there is mesenchymal and angiogenic activation. Blood vessels and MSCs are recruited to the injury site and proliferate. Afterward, MSCs differentiate into either chondrocytes or osteoblasts. Mesenchymal cells differentiate into osteoprogenitors and then proliferate and differentiate into osteoblasts beginning the production and also secretion of osteoid, followed by mineralization, a process termed intramembranous ossification. On the other hand, chondrocytes proliferate and mineralize, and next bone tissue is deposited on the cartilage matrix through a process termed endochondral ossification. Both processes are completed by remodeling the bone to restore normal shape and function.

Most of the approaches of bone tissue engineering use bone marrow-derived cells that are easily accessible, can differentiate into chondrocytes and osteoblasts* in vitro*, and seem to be an ideal autologous cell type [41–44]. Other autologous cell types such as adipose-derived cells, which are also very accessible and possess osteogenic and chondrogenic potential* in vitro*, represent lately a very attractive source.

Adipose-derived stromal cells (ADSCs) are a very useful stem cell population, as they are abundant and can be easily acquired and isolated. However, at the clonal level, only 21% of the population of plastic-adherent ADSCs clones are determined to be tripotent with an additional 31% and 29% exhibiting bipotent and unipotent features, respectively [45]. Interestingly, only 48% of the clones are osteogenic, which means that the surface marker prognostic for osteogenic potency would improve the efficacy of these cells for bone tissue engineering.

Stem cell-based bone tissue engineering with ADSCs has shown great promise for the treatment of large bone deficits. By FACS, a CD105^low^ cells subpopulation with enhanced osteogenic differentiation has been identified. Using single-cell transcriptional analysis, it was found that expression patterns of the cell surface receptor endoglin (CD105) were closely associated with the osteogenic potential of ADSCs (Table 1). By combining microfluidic analysis with FACS, compared with CD105^high^ and unsorted cells, CD105^low^ ADSCs were found to be capable of enhanced osteogenic differentiation [46]. The isolation of ADSCs negative for CD105 was required to form an osteogenic population. This approach was based on previous studies which demonstrated that CD105^−^ ADSCs possess enhanced adipogenic and osteogenic potential, probably due to the reduced TGF-*β*/SMAD2 signaling [46, 47].

Additionally, Leyva-Leyva et al. (2013) positively selected the surface markers CD73, CD44, and CD105 from human amniotic membrane by FACS [18]. Two subpopulations with dissimilar osteoblastic differentiation potential were isolated: CD44^+^/CD73^+^/CD105^+^ (CD105^+^) and CD44^+^/CD73^+^/CD105^−^ (CD105^−^). Using* in vitro *analysis, it was found that the CD105^−^ MSCs subpopulation was associated with more effective calcium deposition. Furthermore, through* in vivo* trials, it was demonstrated that grafts containing CD105^−^ promoted adequate graft integration, improved host vascular infiltration, and showed efficient repair through intramembranous ossification (Table 1). By contrast, grafts containing CD105^+^ showed abundant fibrocartilaginous tissue and deficient endochondral ossification [48].

CD105^+^ and CD105^−^ represent independent subpopulations that maintain their properties upon several passages. In addition to the enhanced osteogenic differentiation potential of the CD105^−^ subpopulation, Anderson et al. reported advantageous immunomodulatory properties. Interestingly, compared to CD105^+^, CD105^−^ murine-derived MSCs suppress the proliferation of CD4^+^ T cells more efficiently [49]. Meanwhile, in humans, the analysis for HLA system profile revealed that the CD105^−^ subpopulation lacked HLA-ABC and HLA-DR (61.65%), which classifies them as nonimmunogenically active [18].

It seems that the surface marker CD105 might predict weak osteogenesis when the source of the isolation is adipose tissue or amniotic membrane [46, 48, 50]; however, when the source is bone marrow, conflicting data have been reported [51].

Aslan et al. found that, in bone marrow, CD105^+^ cells displayed enhanced* in vitro* osteogenic differentiation [52]. Likewise, Jarocha et al. reported that expanded CD105^+^ populations possess higher expression levels for RUNX2 and OCN (early and late osteogenic molecular markers, resp.) [53].

Dennis et al. found that there was good correlation between* in vitro* mineralization and* in vivo* osteogenesis of CD105^+^ cells [54]. Interestingly, these authors also observed a correlation between* in vivo* bone scores with the presence of CD105^+^ cell, suggesting that specific subpopulation seems to be a key aspect in predicting the osteogenic potential of cells

A second cell surface receptor was also found to correlate with the expression of osteogenic markers independent of CD105. CD90 (Thy-1) was originally discovered as a thymocyte antigen, which could be useful to identify and isolate ADSCs subpopulations. CD90^high^ ADSCs had greater reprogramming capacity than CD90^low^ ADSCs, suggesting that ADSCs have heterogeneous subpopulations [55]. Moreover, Hosoya et al. evaluated the capacity of rat CD90^high^ and CD90^low^ subodontoblastic dental pulp stem cells to differentiate into hard tissue-forming cells in response to bone morphogenetic protein-2 stimulation and observed that CD90^high^ had accelerated ability to mineralize* in vitro* and* in vivo* (Table 1) [56].

CD90 and CD105 have been identified as early MSCs markers present on both BM-MSCs and ADSCs. Chung et al. demonstrated that, compared with CD90^−^ or unsorted cells, CD90^+^ subpopulation isolated from human adipose tissue has enhanced osteogenic potential* in vitro* and* in vivo*; in fact, the authors proposed CD90 as a better surface marker to isolate cells with osteogenic potential [57, 58]. Murine-derived ADSCs were sorted for the expression of the surface markers CD90 and CD105 using flow cytometry. ADSCs were sorted into four groups: CD90^+^/CD105^−^, CD90^+^/CD105^+^, CD90^−^/CD105^+^, and CD90^−^/CD105^−^, in which CD90^+^/CD105^−^ and CD90^+^/CD105^+^ cells had robust osteogenic potential and displayed mineralized nodules, whereas strong expression of CD105 might predict weak osteogenesis [50].

Consistent findings indicate that the absence of CD105 and the expression of CD90 surface markers characterize subpopulations with increased efficiency of differentiation into osteogenic lineage.

It has been advised not to discard the possibility of including other markers as part of an osteogenic profile analysis. Recently, the expression of the human embryonic stem cells marker SSEA-4 in a subpopulation of human adipose tissue (SSEA-4^+^ hASCs) has been reported. The subpopulation has the ability to differentiate into osteogenic lineages but also into endothelial lineages, which represents a useful approach to obtain these two cell types from the source and consequently is relevant for bone tissue engineering applications (Table 1) [59, 60].

### 3.2. Chondrogenic Differentiation from Mesenchymal Stem Cell Subpopulations

For clinical success, MSCs must be held in the area of injury and produce extracellular matrix in a physiological context, where low nutrient conditions produced by avascularity, nutrition, and waste production are prevalent. Certain MSCs subpopulations are more resilient to metabolic challenge than others.

Chondrogenic differentiation of BM-MSCs has been extensively studied* in vitro* in micromass pellet, which promotes cell condensation, aside from cell-cell and cell-extracellular matrix (ECM) connections [61, 62]. Consequently, cells progress into a highly proliferative stage to produce typical components of the cartilaginous matrix (collagen type 2, collagen type 9, aggrecan, and cartilage oligomeric matrix protein). Lastly, cells become round and then go through hypertrophy expressing collagen type X and MMP13 [63–67].

Cartilage is susceptible to damage and has a reduced capacity for regeneration. Procedures committed to recruit stem cells from BM by penetration to the subchondral bone have been commonly used to treat localized cartilage defects [68]. More recently, autologous chondrocyte implantation has been introduced [69]. Research on cartilage tissue engineering in recent years has focused on the use of adult MSCs as an alternative source of autologous chondrocytes [70].

MSCs can differentiate into chondrocytes and fibrochondrocytes, resulting in a combination of cartilaginous fibrous and hypertrophic tissues, whereby the clinical success lasted for a short time because these cells do not possess functional mechanical properties [71]. Conversely, compared to MSCs derived from BM, MSCs from synovial tissue have been revealed to enhance chondrogenic potential and diminish the hypertrophic differentiation [72, 73].

Fickert et al. sorted a triplicate positive subpopulation from the synovial membrane (SM) of patients with osteoarthritis (CD9^+^/CD90^+^/CD166^+^). In the micromass of sorted cell cultures, Col2 was located predominantly in the inner areas, indicating that the subpopulation of SM-derived cells has the capacity to differentiate efficiently towards the chondrogenic lineage (Table 2). However, no major differences between sorted and unsorted SM cells were evidenced [74, 75].

In 2010, Arufe et al. analyzed the chondrogenic potential of subpopulations of human synovial membrane MSCs sorted for CD73, CD106, and CD271 markers. Compared with CD106^+^ and CD271^+^ subpopulations, CD73^+^ cells evidenced the highest expression of* SOX9* (a key transcription factor that is necessary for early chondrogenesis), aggrecan, and COL2A1 at day 46 of chondrogenic induction. However, the CD73^+^ cells also showed the expression of COL10A1, indicating the presence of hypertrophy during differentiation [76].

More recently, in 2013, it was reported that the isolation of a different SM subpopulation based on surface markers CD73 and CD39 displayed consistent dynamics over passaging. The CD73^+^CD39^+^ cell subpopulation displayed higher expression levels of* SOX9* and a significantly greater chondrogenic potency than the CD73^+^CD39^−^ cell subpopulation (Table 2) [77].

Regarding the CD271 surface marker, compared to the other subpopulations, the CD271^+^ subpopulation expressed the highest levels of COL2 staining. Spheroids formed from CD271^+^ and CD73^+^ subpopulations from normal human synovial membranes that imitate the native cartilage extracellular matrix more closely than CD106^+^ MSCs, with the result that both are excellent candidates to use in cartilage tissue engineering [76].

Hermida-Gómez et al. strengthened this finding, showing that, during spontaneous cartilage repair, CD271^+^ provides higher quality chondral repair than the CD271^−^ subpopulation. The implantation of MSCs CD271^+^ provided such benefits as greater filling of the chondral defect and improved integration between the repair tissue and native cartilage (Table 2) [78].

Meanwhile, Arufe et al. reported the isolation by a magnetic separator of a CD105^+^ subpopulation from human synovial membrane. These researchers evidenced a homogeneous cellular culture, which expressed Sox9 and had the ability to develop spheroids after 7 days in the presence of chondrogenic medium (Table 2). Interestingly, the extracellular matrix produced is rich in Col2 and showed no evidence of fibrocartilage tissue. The analysis of the CD105^−^ subpopulation was not reported [79].

Tendon-derived progenitor cells (TPCs) from mice contained two subpopulations: one positive and one negative for CD105. Compared to the* in vitro* case with CD105^+^, the CD105^−^ negative cells showed superior chondrogenic potential, and it was proposed that differences in the capability of chondrogenic differentiation are due to different modes of smad1/5 and smad2/3 signaling activation as a result of TGF*β*s (Table 2) [80].

Various parameters have been considered in hMSCs' chondrogenic differentiation. In particular, it has been evidenced that hMSCs' expansion* in vitro* required FGF-2 and IGF-1 to enhance the proliferative and chondrogenic potential [81–83]. A highly efficient strategy is based on the preselection during the expansion phase of the MSCs by adding growth factors. In 2013, Hagmann et al. reported that FGF-2 suppressed CD146 expression and significantly improved chondrogenic differentiation [84]. Despite the observations from the preselection with FGF-2 and resulting suppression of CD146, in 2014, these researchers demonstrated that, compared to control MSCs, CD146^+^ FACS-sorted cells showed significantly increased GAG/DNA content after chondrogenic differentiation [85]. It should be noted that subpopulations, such as CD146^+^ from human umbilical cords, not only provide more efficient cartilage regeneration process but also provide an anti-inflammatory protective microenvironment resulting from decreased expression of IL-6 (Table 2) [86].

## 4. Conclusions

The current review highlights recent findings in the isolation and characterization of MSCs subpopulations and the potential applications for osteogenic or chondrogenic differentiation.

It was evident that the source of the MSCs subpopulation had an effect on the differentiation potential, and certainly the use of strategies based on the coexpression of more than one surface marker improves the purity of the isolated MSCs population.

These findings indicate that the absence of the CD105 surface marker characterizes subpopulations with improved osteogenesis when the source of isolation is adipose tissue or amniotic membrane. Furthermore, subpopulations expressing CD271 or CD146 markers appear to provide higher quality for chondral repair.

An accurate selection of the subpopulation puts forward possibilities suitable for preclinical and clinical studies and determines the safest and most efficacious regeneration process.

## Figures and Tables

**Table 1:** MSC subpopulations with enhanced osteogenic differentiation.

Subpopulation markers	Isolation method	Source	Reference
CD105^low^	FACS	hADSCs	Levi et al. 2011 [46]

CD44^+^/CD73^+^/CD105^−^	FACS	AM-hMSCs	Leyva-Leyva et al. 2015 [48]

CD105^−^	Microbeads	mADSCs	Anderson et al. 2013 [49]

CD105^+^	Microbeads	BM hMSCs	Aslan et al. 2006 [52]
			Dennis et al. 2007 [54]
			Jarocha et al. 2008 [53]

CD90^high^	FACS	Rat dental pulp cells	Hosoya et al. 2012 [56]

CD90^+^	FACS	hADSCs	Chung et al. 2013 [58]
	FACS	mADSCs	Yamamoto et al. 2014 [50]

SSEA-4^+^	Magnetic beads	hADSCs	Mihaila et al. 2013 [59]

**Table 2:** MSC subpopulations with enhanced chondrogenic differentiation.

Subpopulation markers	Isolation method	Source	Reference
CD9^+^/CD90^+^/CD166^+^	FACS	SM	Fickert et al. 2003 [74]

CD271^+^	FACS	SM	Arufe et al. 2010 [76]
	Magnetic beads	SM	Hermida-Gómez et al. 2010 [78]

CD73^+^CD39^+^	FACS	SM	Gullo and De Bari 2013 [77]

CD105^+^	Magnetic beads	SM	Arufe et al. 2009 [79]

CD105^−^	FACS	mTPCs	Asai et al. 2014 [80]

CD146^+^	FACS	BM	Hagmann et al. 2013 [84]
	Magnetic beads	HU-MSCs	Wu et al. 2016 [86]

## Abbreviations

ACPC: Articular cartilage progenitor cells
ADSCs: Adipose-derived stromal cells
ALP: Alkaline phosphatase
AM: Amniotic membrane
AT: Adipose tissue
BM: Bone marrow
BM-MSCs: Bone marrow mesenchymal stem cells
CD: Cluster of differentiation
CFU-Fs: Colony forming unit-fibroblastoids
DPSCs: Dental pulp stem cells
ECM: Extracellular matrix
FACS: Fluorescence-activated cell sorting
ISCT: International Society for Cellular Therapy
HU-MSCs: Human umbilical mesenchymal stem cells
MSCs: Mesenchymal stem cells
PDLPs: Periodontal ligament progenitor cells
PDLSCs: PDL stem cells
PSC: Perivascular stem cells
OA: Osteoarthritis
SHED: Stem cells from human exfoliated deciduous teeth
SM: Synovial membrane
TPCs: Tendon-derived progenitor cells
VCAM: Vascular cell adhesion molecule.
